# Is robotic distal pancreatectomy better than laparoscopic distal pancreatectomy after the learning curve? A systematic review and meta-analysis

**DOI:** 10.3389/fonc.2022.954227

**Published:** 2022-08-29

**Authors:** Chuwen Chen, Jing Hu, Hao Yang, Xuejun Zhuo, Qiuping Ren, Qingbo Feng, Miye Wang

**Affiliations:** ^1^ Department of Liver Surgery and Liver Transplantation Centre, West China Hospital, Sichuan University, Chengdu, China; ^2^ Department of Health Management Centre, West China Fourth Hospital of Sichuan University, Chengdu, China; ^3^ Engineering Research Centre of Medical Information Technology, Ministry of Education, West China Hospital, Sichuan University, Chengdu, China; ^4^ Information Technology Centre, West China Hospital of Sichuan University, Chengdu, China

**Keywords:** minimally invasive surgery, robotic distal pancreatectomy, laparoscopic distal pancreatectomy, Da Vinci, meta-analysis

## Abstract

**Aim:**

The aim of this study was to compare the safety and overall effect of robotic distal pancreatectomy (RDP) to laparoscopic distal pancreatectomy (LDP) after the learning curve, especially in perioperative outcome and short-term oncological outcome.

**Methods:**

A literature search was performed by two authors independently using PubMed, Embase, and Web of Science to identify any studies comparing the results of RDP versus LDP published until 5 January 2022. Only the studies where RDP was performed in more than 35 cases were included in this study. We performed a meta-analysis of operative time, blood loss, reoperation, readmission, hospital stay, overall complications, major complications, postoperative pancreatic fistula (POPF), blood transfusion, conversion to open surgery, spleen preservation, tumor size, R0 resection, and lymph node dissection.

**Results:**

Our search identified 15 eligible studies, totaling 4,062 patients (1,413 RDP). It seems that the RDP group had a higher rate of smaller tumor size than the LDP group (MD: −0.15; 95% CI: −0.20 to −0.09; *p* < 0.00001). Furthermore, compared with LPD, RDP was associated with a higher spleen preservation rate (OR: 2.19; 95% CI: 1.36–3.54; *p* = 0.001) and lower rate of conversion to open surgery (OR: 0.43; 95% CI: 0.33–0.55; *p* < 0.00001). Our study revealed that there were no significant differences in operative time, overall complications, major complications, blood loss, blood transfusion, reoperation, readmission, POPF, and lymph node dissection between RDP and LDP.

**Conclusions:**

RDP is safe and feasible for distal pancreatectomy compared with LDP, and it can reduce the rate of conversion to open surgery and increase the rate of spleen preservation, which needs to be further confirmed by quality comparative studies with large samples.

**Systematic Review Registration:**

https://www.crd.york.ac.uk/PROSPERO/#recordDetails.

## Introduction

Laparoscopic distal pancreatectomy (LDP) was firstly reported by Cuschieri in 1994 (1). In recent years, LDP was favored for being minimally invasive, reducing surgical morbidity and intraoperative blood loss, having a rapid postoperative recovery rate, and providing a high comfort level to patients (2–5). Robotic distal pancreatectomy (RDP) was first reported in 2003 (6), compared with the conventional laparoscopic procedures, and RDP overcomes some of the disadvantages (limited range of motion, reliance on two-dimensional imaging, reduced dexterity, fulcrum effect, natural tremors, poor surgeon ergonomics, and difficulty in vascular control), which made minimally invasive surgery popular in pancreas surgery (7). Although RDP has many advantages over LDP, overcoming this learning curve requires a relatively long training period for surgeons. It is well known that surgeons’ experience and performance play an important role in patient outcomes, which can lead to bias. Murtaza Shakir et al.’s study showed that the learning curve for RDP was 40 cases (8). However, Benrizi et al.’s study revealed that the learning curve was completed after 11 operations (9). Furthermore, when surgeons do not overcome the learning curve, surgical outcomes are often unsatisfactory, even at high-volume centers. As far as we know, there was no study comparing the perioperative and short-term oncological outcomes between RDP and LDP to avoid bias. Therefore, we conducted a systematic review and meta-analysis of studies that compare RDP and LDP after the learning curve by good quality articles.

## Methods

### Materials and methods

This review was registered with PROSPERO (CRD42021268106) and reported with reference to the PRISMA guidelines (10).

### Search schedule

An electronic search of the PubMed/MEDLINE, EMBASE, and Cochrane Library database for articles relating to RDP and LDP before 5 January 2022 was performed by three independent investigators (CC, QF, and MW). The search terms were the following: “robotic surgery” OR “robot-assisted” OR “robot” OR “robotic” OR “Da Vinci” AND “laparoscopic surgery” OR “laparoscope” AND “distal pancreatectomy”, either individually or in combination. The references of included articles were also screened manually for a comprehensive search.

### Study selection

Two independent researchers (CC and QF) independently reviewed current articles to check the eligibility for inclusion, and the third author (JH) participated in the evaluation of controversial articles. Retrospective and prospective cohort studies, cross-sectional studies, and randomized controlled trials with a reported RDP of greater than or equal to 35 cases were considered for inclusion. The latest study and PSM study were included to analysis, when the duplicate studies from the same institutions. Studies exclusion criteria: (I) non-English language articles; (II) no comparative analysis between RDP and LDP; (III) pediatric and pregnant women as participants; (IV) multicenter studies; and (V) outcomes of the following were not reported in the literature: reoperation, operation time, readmission, blood loss, hospital stay, tumor size, blood transfusion, R0 rate, conversion rate, lymph node harvested, overall complications, major complications, ROPF, and spleen preservation rate.

### Data extraction and quality assessment

Literature characteristics and patient characteristics (including operative time, mean age, blood loss, blood transfusion, tumor size, overall complication, major complication, hospital stay, R0 rate, blood transfusion, reoperation, readmission, POPF, and number of harvested lymph nodes) were extracted by two authors (CC and QF) into a unified datasheet. We consulted a third observer (MW) when there was an ambiguity in the study. A quality assessment of every included study was adopted using the Newcastle–Ottawa Scale (NOS), and NOS ≥6 was considered as being of high quality (11).

### Statistical analysis

Statistical analyses were performed by Review Manager Software (RevMan5.3; The Nordic Cochrane Centre, The Cochrane Collaboration, Copenhagen, Denmark). The 95% confidence interval (CI) and mean difference (MD) were used for continuous data. For dichotomous data, the pooled odds ratio (OR) with 95% CI was used. The method reported by Hozo et al. was used to convert medians and range values into means and standard deviations (12). Funnel plots and the *I* ^2^ index were respectively used to assess potential publication bias and statistical heterogeneity. When heterogeneity was low or moderate (*I* ^2^ < 50%), the fixed-effects model (FEM) was adopted. Meanwhile, for the study with high heterogeneity (*I* ^2^ ≥ 50%), the random-effects model (REM) was considered.

## Results

### Literature results

**Figure 1:**
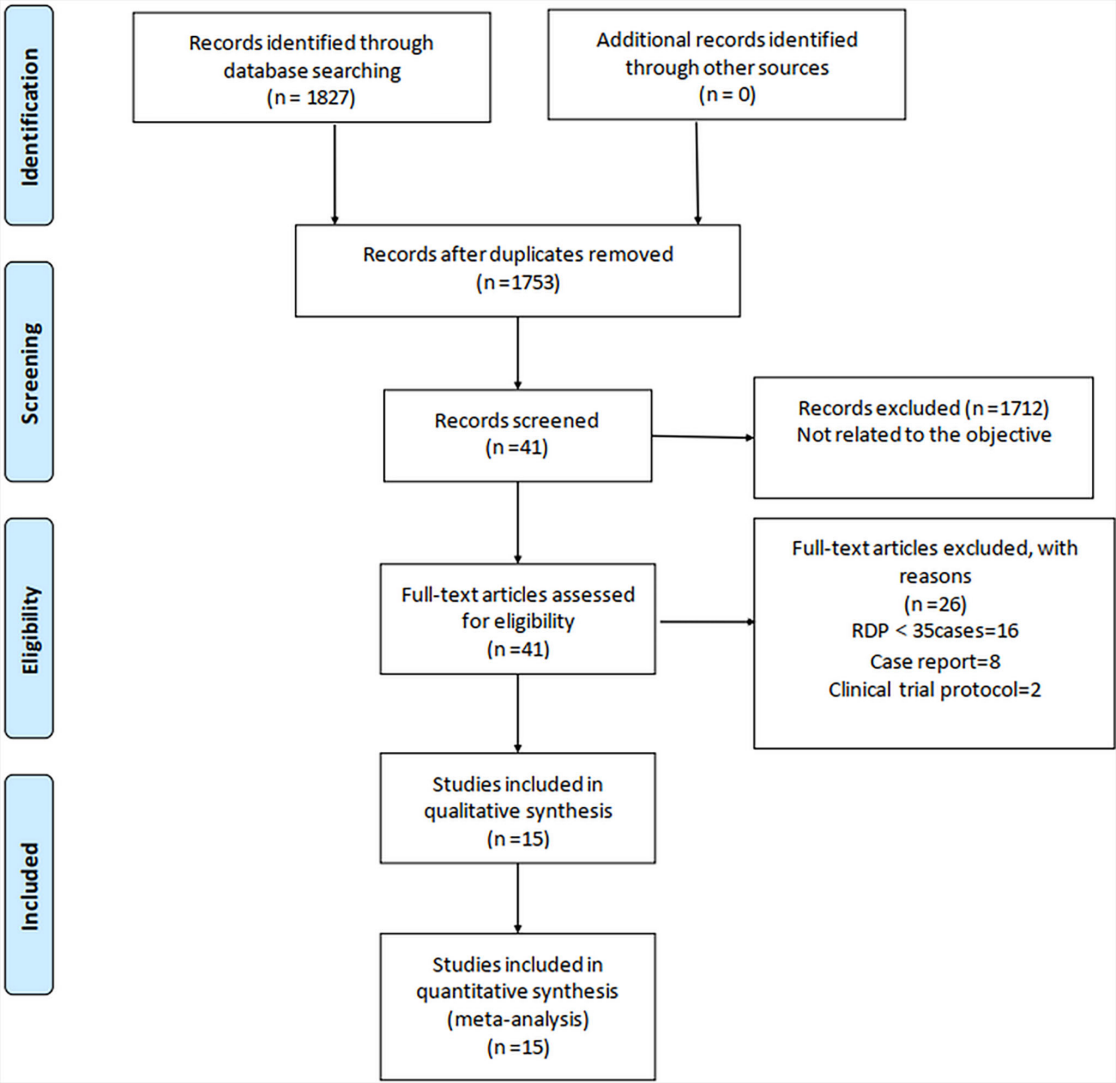
Flowchart of study identification and selection.

In total, 1,827 relevant English articles were initially identified for evaluation. After scanning for inclusion criteria, a total of 4,602 patients [15 studies (7, 13–26)] were included this study; 1,413 and 3,189 patients underwent RDP and LDP, respectively. A flow diagram (**Figure 1**) shows our analysis scheme, and **Table 1** reports the summary of the key characteristics and NOS for included articles.

**Table 1:** Characteristics of included studies.

Author-year	Type of study	Period	Country	Patients		Age (years) mean ± SD		Gender (M/F)		BMI (kg/m^2^)		NOS
				RDP	LDP	RDP	LDP	RDP	LDP	RDP	LDP	
Adam-2015	Retrospective	2010–2011	USA	61	474	65 ± 14	64 ± 13	28/33	248/226	NA	NA	8
Chen-2015	Retrospective	2005–2014	China	69	50	56.2 ± 13.3	56.5 ± 15	23/46	18/32	24.6 ± 2.8	24.6 ± 3.0	8
Lee-2015	Retrospective	2000–2013	USA	37	131	58 ± 11.1	58 ± 15	10/27	57/74	28.7	28.2	8
Liu-2017	PSM	2011–2015	China	102	102	48.10 ± 15.59	49.62 ± 15.24	34/68	47/55	NA	NA	8
Xourafas-2017	Retrospective	2014–2014	USA	200	694	62 (22–88)	62 (19–89)	83/117	275/419	28.8 (15–55)	28.4 (17–59)	8
Zhang-2017	Retrospective	2010–2017	China	43	31	47.9 ± 10.5	48.7 ± 12.3	20/23	12/19	23.9 ± 3.2	23.3 ± 2.7	7
Qu-2018	PSM	2011–2015	China	35	35	58.1 ± 11.1	57.8 ± 11.4	22/11	22/11	24.46 ± 3.30	24.08 ± 3.73	8
Marino-2018	Case-match	2014–2017	Italy	35	35	59.3 (40–73)	58.5 (34–69)	20/15	19/16	NA	NA	7
Raoof-2018	Retrospective	2010–2013	USA	99	605	NA	NA	45/54	322/283	NA	NA	7
Lyman-2018	Retrospective	2008–2017	USA	108	139	56.3 ± 16.1	59.5 ± 15.5	46/62	75/64	29.3 ± 6.5	29.0 ± 8.5	7
Hong-2019	Retrospective	2015–2017	South Korea	46	182	51.2 ± 13.8	60.2 ± 13	32/14	88/94	24.9 ± 4.1	24.6 ± 3.2	7
Pastena-2020	PSM	2011–2017	Italy	37	66	50 (39–65)	53(40–62)	13/24	20/46	24 (22–27)	24 (21–28)	8
Franco-2021	Case-match	2008–2020	Italy	35	35	60.4 ± 13.2	63.9 ± 16.9	11/24	17/18	26.2 ± 4.7	26.0 ± 5.5	7
Kwon-2021	PSM	2015–2020	South Korea	104	208	50.62 ± 13.65	51.23 ± 14.52	35/69	72/136	24.05 ± 3.86	24.06 ± 3.55	8
Lof-2021	PSM	2011–2019	UK	402	402	57 ± 15	57 ± 14	165/237	158/244	25.4 ± 4.6	25.9 ± 5.0	8

RDP, robotic distal pancreatectomy; LDP, laparoscopic distal pancreatectomy; M/F, male/female; SD, standard deviation, BMI, body mass index; NA, not available; PSM, propensity score matching.

### Perioperative outcomes

To evaluate the perioperative outcomes, we compared the operative time, hospital stay, blood loss, blood transfusion, overall complication rates, major complications, postoperative pancreatic fistula, R0 rate, conversion to open surgery, spleen preservation, POPF, reoperation, and readmission.

### Operative time

**Figure 2:**
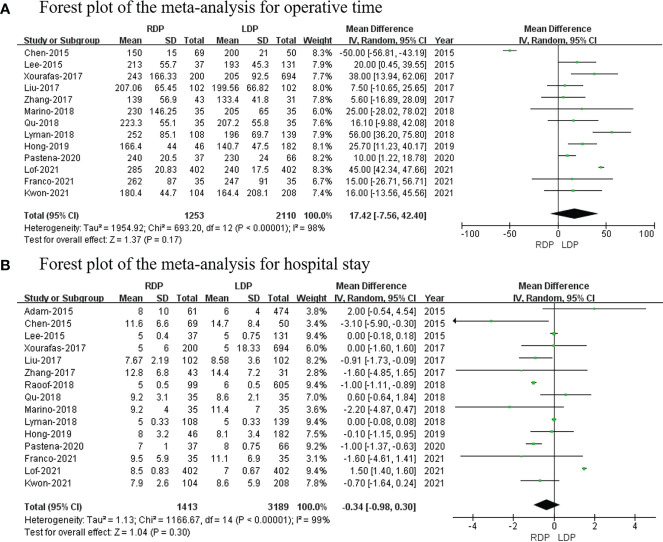
Forest plot of the meta-analysis for **(A)** operative time and **(B)** hospital stay.

Thirteen studies (7, 14–19, 21–26) (1,253 and 2,110 patients from the RDP group and LDP group, respectively) reported operative times. This meta-analysis revealed that there was no significant difference between the two groups (WMD: 17.42 min; 95% CI: −7.56–42.40; *p* = 0.17) with high heterogeneity (*I* ^2^ = 98%; shown in **Figure 2A**).

### Hospital stay

All studies (7, 13–26) with a total of 4,602 patients (1,413 patients underwent RDP; 3,189 patients underwent LDP) investigated hospital stay. This meta-analysis showed no difference in hospital stay between the two groups (*p* = 0.30; 95% CI: −0.98 to 0.30; shown in **Figure 2B**).

### Blood loss

**Figure 3:**
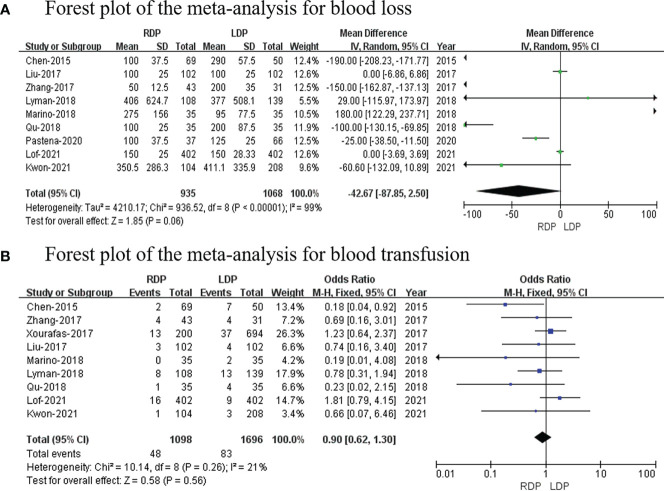
Forest plot of the meta-analysis for **(A)** blood loss and **(B)** blood transfusion.

Nine studies (14, 16, 18, 19, 21–24, 26) reported the estimated blood loss volume, and this meta-analysis revealed no difference in blood loss (MD: −42.67 ml; 95% CI: −87.85 to 2.50; *p* = 0.06) with high heterogeneity (*I* ^2^ = 99%; shown in **Figure 3A**).

### Blood transfusion

Blood transfusion data were available in nine studies (14, 16–19, 21, 22, 24, 26). This study revealed that blood transfusion rate was not different between RDP and LDP (OR: 0.90; 95% CI: 0.62–1.30; *p* = 0.56) with low heterogeneity (*I* ^2^ = 21%; shown in **Figure 3B**).

### Overall complication rates

**Figure 4:**
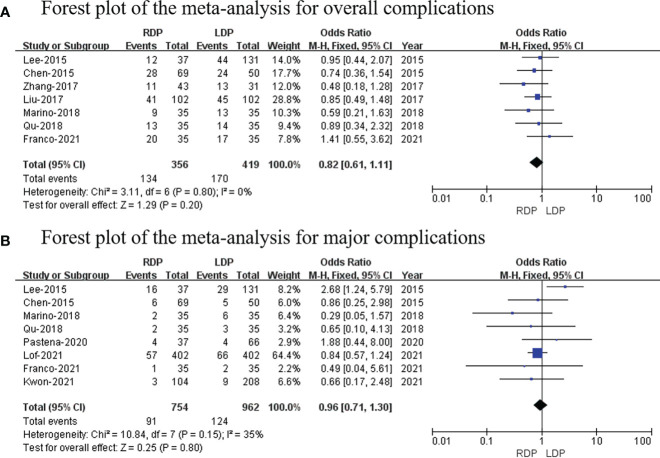
Forest plot of the meta-analysis for **(A)** overall complications and **(B)** major complications.

Seven studies (14–16, 18, 19, 22, 25) (a total of 775 patients; 356 and 419 patients from the RDP group and LDP group, respectively) reported postoperative complications. Our study revealed that there was no significant difference in two groups (OR: 0.82; 95% CI: 0.61–1.11; *p* = 0.20) with no heterogeneity (*I* ^2^ = 0%; **Figure 4A**).

### Major complications

Eight studies (14, 15, 19, 22–26) (754 and 962 patients from the RDP group and LDP group, respectively) recorded major complications. Grade III to V complications based on the Clavien–Dindo classification were considered as major complications (27). No significant differences in major complications were observed between these two groups (OR: 0.96; 95% CI: 0.71 to 1.30; *p* = 0.80) with low heterogeneity (*I* ^2^ = 35%; **Figure 4B**).

### Postoperative pancreatic fistula

**Figure 5:**
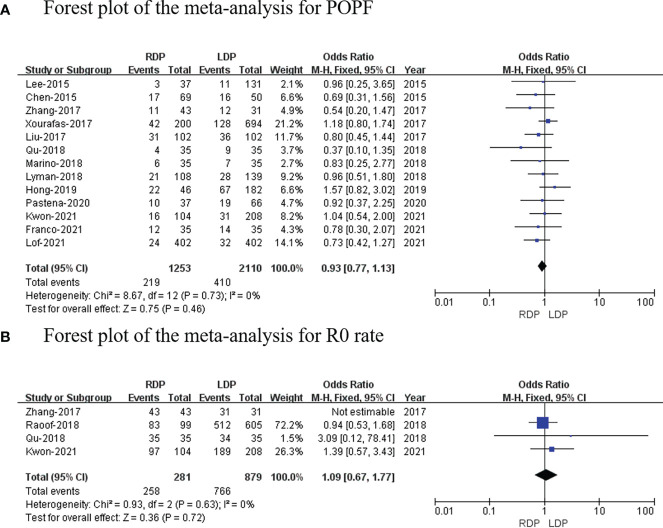
Forest plot of the meta-analysis for **(A)** postoperative pancreatic fistula and **(B)** R0 rate.

In total, 13 studies (3,363 patients) reported the incidence rate of POPF (7, 14–19, 21–26). Our study found that there was no significant difference in POPF rate between the two groups (OR: 0.93; 95% CI: 0.77 to 1.13; *p* = 0.46) with no heterogeneity (*I* ^2^ = 0%) (**Figure 5A**).

### R0 resection rate

In total, four studies (281 and 962 patients from the RDP group and LDP group, respectively) reported the R0 resection rate (18–20, 26). No significant differences in R0 resection rate were observed between the two groups (OR: 1.09; 95% CI: 0.67–1.77; *p* = 0.72) with no heterogeneity (*I* ^2^ = 0%; **Figure 5B**).

### Conversion to open surgery

**Figure 6:**
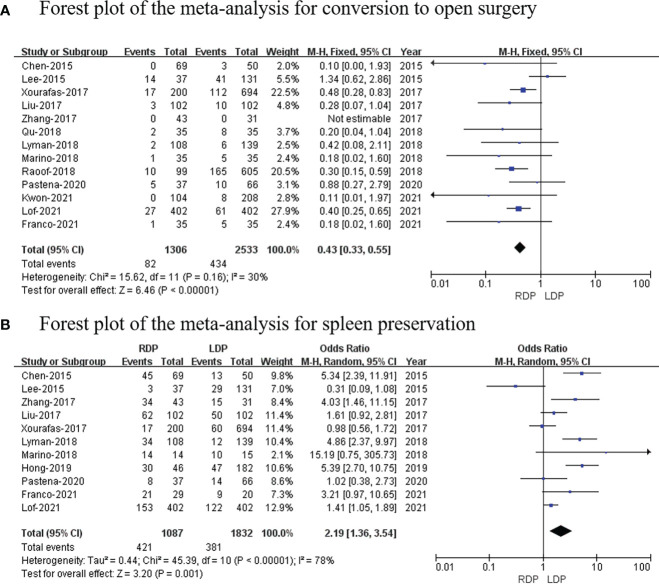
Forest plot of the meta-analysis for **(A)** conversion to open surgery and **(B)** spleen preservation.

Thirteen studies (14–26) (1,306 patients underwent RDP and 2,533 patients underwent LDP) reported conversion to open surgery, and this meta-analysis indicated that the higher conversion rate was observed in the RDP group (OR: 0.43; 95% CI: 0.33 to 0.55; *p* < 0.00001) with low heterogeneity (*I* ^2^ = 30%; **Figure 6A**).

### Spleen preservation

Eleven studies (7, 14–19, 21–25) with a total of 2,919 patients reported the spleen preservation rate, and this study found that there was a significant higher spleen preservation rate in the RDP group (OR: 2.19; 95% CI: 1.36 to 3.54; *p* = 0.001) with high heterogeneity (*I* ^2^ = 78%; **Figure 6B**).

### Reoperation

**Figure 7:**
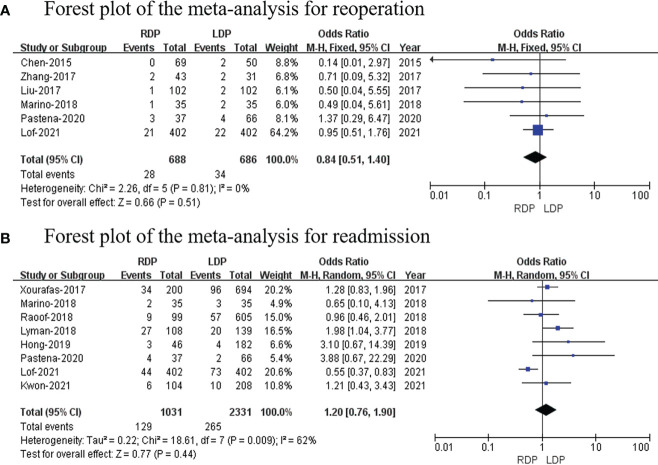
Forest plot of the meta-analysis for **(A)** reoperation and **(B)** readmission.

The data of six studies (14, 16, 18, 22–24) (with a total of 1,374 patients) that assessed reoperation were pooled, and our analysis revealed no difference in reoperation (OR: 0.84; 95% CI: 0.51 to 1.40; *p* = 0.51) with no heterogeneity (*I* ^2^ = 0%; **Figure 7A**).

### Readmission

Eight studies (7, 17, 20–24, 26) that included 3,362 patients assessed readmission. The study showed that there was no difference in readmission (OR: 1.20; 95% CI: 0.76–1.90; *p* = 0.74) with high heterogeneity (*I* ^2^ = 62%; **Figure 7B**).

### Short−term oncological outcomes

#### Tumor size

**Figure 8:**
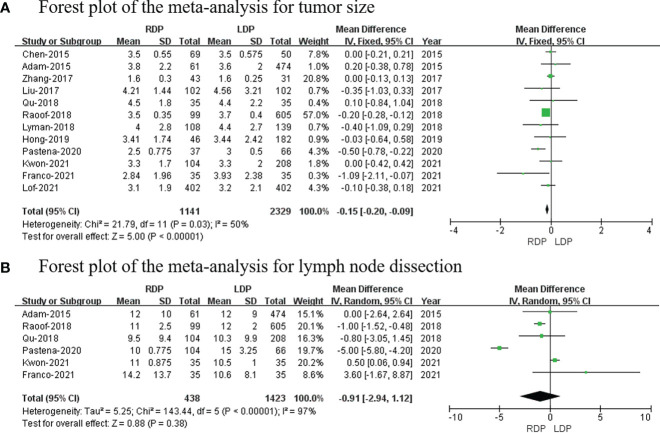
Forest plot of the meta-analysis for **(A)** tumor size and **(B)** lymph node dissection.

Twelve studies (7, 13, 14, 16, 18–21, 23–26) that included 3,470 patients reported the tumor size, and the results of the meta-analysis showed that RDP has a smaller tumor size than the LDP group (MD: −0.15; 95% CI: −0.20 to −0.09; *p <*0.00001) with high heterogeneity (*I* ^2^ = 50%; **Figure 8A**).

### Lymph node dissection

Six studies (13, 19, 20, 23, 25, 26) including 1,861 patients reported lymph node dissection; meanwhile, this meta-analysis revealed that there was no difference in lymph node dissection (MD: −0.91; 95% CI: −2.94 to 1.12; *p* = 0.38) with high heterogeneity (*I* ^2^ = 97%; **Figure 8B**).

### Publication bias

**Figure 9:**
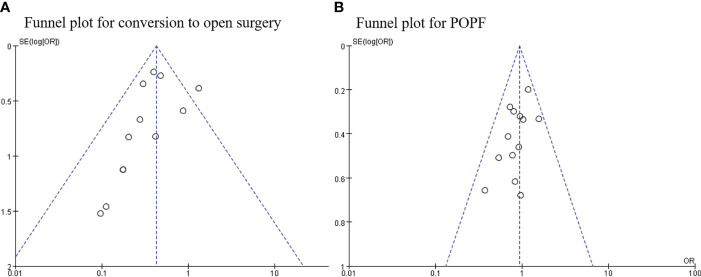
**(A)** Funnel plot for conversion to open surgery and **(B)** postoperative pancreatic fistula.

Begg’s funnel plot was used to assess publication bias for each outcome. As shown in the funnel plots,conversion to open surgery (**Figure 9A**) and POPF (**Figure 9B**) all studies are within the 95% CI, indicating no publication bias.

## Discussion

The special anatomy, complicated vascular variations, and various postoperative complications make pancreatic resection a challenging surgical procedure (25). As minimally invasive surgery development, LDP and RDP have developed rapidly in recent years (28, 29). Previous studies have demonstrated that the LDP has the same safety and efficacy as open surgery (30, 31). Meanwhile, the LDP is more minimally invasive than traditional surgery (30, 31). Our study was designed to compare the clinical outcomes of RDP versus LDP after the learning curve. As we all know, the surgeons’ experience and performance play an important role in patient outcomes. The surgical results during the learning curve are not satisfactory, even at high-volume centers.

At present, some studies have reported the learning curve of RDP, but the heterogeneity of the outcome of these studies was significant (9, 32–35). A study by Murtaza Shakir et al. found the learning curve for RDP to be 40 cases (8). Additionally, two single-surgeon centers reported the learning curve for RDP: the RDP learning curve was reported to be five surgeries in a series of 43 by Takahashi et al., and the RDP learning curve was 10 surgeries by Napoli et al. (33, 36). However, two other multi-surgeon groups published reports in the literature: Benrizi et al. reported only 11 surgeries, while Shyr et al. reported 37 surgeries (9, 34). A study by Amr et al. determined that the RDP learning curve is 20–40 surgeries, with operating time being the most significant factor. In view of the above, we finally set RDP ≥ 35 as the criteria for passing the learning curve. Currently, the surgeons who performed RDP have gone through the LDP learning curve.

Finally, 15 retrospectives studies (4,602 patients) were incorporated into this study to compare the perioperative outcomes and oncologic outcomes between RDP and LPD after the learning curve. Our study found that RDP had a lower rate of conversion to open than LDP, which was consistent with the current mainstream clinical studies (17, 24, 28). Meanwhile, our study found a higher rate of spleen preservation in the RDP group than in the LDP group, which was consistent with the current mainstream publishing clinical studies (32, 37). We think that this may be explained by the fact that RDP has several technical advantages over laparoscopic techniques that make it potentially advantageous. These include a three-dimensional surgical view, motion scaling, tremor filtration, improved surgeon ergonomics, and the wide range of motion of the articulating instruments (7). These advantages lead to RDP having an upper hand in dealing with the special anatomy structure and complicated vascular variation, which reduced the rate of splenectomy (5, 34, 35). Undoubtedly, surgical technique and experience, as well as various patient factors (tumor types, tumor location, vascular invasion, surgical schedule, etc.), play a major role in spleen preservation (35).

In our study, the RDP group had a smaller tumor size than the LDP group, which may be related to the RDP patients with benign or early-stage diseases. Surgeons tended to select LDP on patients with malignancy because it was more familiar to them than RDP, although Al Abbas et al. reported that RDP has a longer operative time and hospital stay (35). In our study, we found that there was no difference in operative time and length of hospital stay between RDP and LDP. This was easily explained in the Al Abbas et al. study that included literature with small samples and wherein the surgeons had not completed the learning stage. Recent studies have shown that the operative time and length of hospital stay were the same between RDP and LDP, as most clinical trials have shown (22, 24, 26). Distal pancreatectomy with negative margin and lymph node dissection are two important prognosis factors (7, 22, 38). Based on the tumor radical effect, our study showed that there was no difference in RDP and LDP lymph node dissections. Similar radical effects can be considered between RDP and LDP, which is consistent with major existing clinical studies (7, 22, 24, 25, 29, 38). Meanwhile, this study shows that no significant difference was observed in reoperation, blood transfusion, readmission, overall complication rates, POPF, and major complications (Clavien–Dindo≥3/4 grade) between the RDP group and LDP group, showing that RDP is as safe as LPD after the learning curve. Overall, a high rate of spleen preservation and low open conversion rates make RDP a safe and feasible alternative to LDP. Undoubtedly, the hospital cost of RDP was a crucial factor limiting the widespread use of RDP. However, the hospital cost was not described in detail in the included literature, and thus we could not perform further analysis. It is believed that with the development and modernization of robot technology and the many obvious advantages of RDP, including reduced cost, RDP will be widely used in the future.

This study included 15 studies to compare safety and efficiency following RDP and LDP. However, there are still several limitations to our study. Firstly, because our study included articles that were retrospective in nature, there may be inherent selection biases. In addition, addition, a short follow-up period in the included literature prevented the assessment of some long-term outcomes (overall survival and disease-free survival). Moreover, some studies had benign conditions included in them, which may affect patient prognosis. Therefore, in order to resolve this problem, we need to conduct larger prospective comparative studies and randomized clinical trials.

## Conclusion

In summary, our meta-analysis found that RDP is a safe alternative to LDP as it is associated with a significant reduction in conversion to open surgery and increased spleen preservation. After the learning curve, RDP is a technically oncologically safe and feasible approach. Because RDP achieved similar outcomes to LDP, it should be the preferred choice. Future higher-quality large-scale comparative studies and long-term follow-up periods are necessary to confirm the safety and efficacy of RPD after the learning curve.

## Data availability statement

The original contributions presented in the study are included in the article/supplementary materials. Further inquiries can be directed to the corresponding author.

## Author contributions

Conception and design: CWC, JH and MYW; Provision of study materials or patients: CWC, QBF and MYW; Collection and assembly of data: CWC, QBF and JH; Data analysis and interpretation: CWC, MYW, XJ Zhuo; Manuscript writing: CWC, MYW, JH and XJZ; Manuscript review: CWC, JH, MYW. Final approval of manuscript: All authors.

## Funding

This work was supported by Model research and application demonstration of hierarchical coordination within health alliance based on a cloud platform (Project No.2020YFS0092).

## Conflict of interest

The authors declare that the research was conducted in the absence of any commercial or financial relationships that could be construed as a potential conflict of interest.

## Publisher’s note

All claims expressed in this article are solely those of the authors and do not necessarily represent those of their affiliated organizations, or those of the publisher, the editors and the reviewers. Any product that may be evaluated in this article, or claim that may be made by its manufacturer, is not guaranteed or endorsed by the publisher.

## References

[1] Cuschieri A . Laparoscopic surgery of the pancreas. J R Coll Surg Edinb (1994) 39(3):178–84. 7932341

[2] Asbun HJ Moekotte AL Vissers FL Kunzler F Cipriani F Alseidi A . The miami international evidence-based guidelines on minimally invasive pancreas resection. Ann Surg (2020) 271(1):1–14. doi: 10.1097/SLA.0000000000003590 31567509

[3] Sharpe SM Talamonti MS Wang E Bentrem DJ Roggin KK Prinz RA . The laparoscopic approach to distal pancreatectomy for ductal adenocarcinoma results in shorter lengths of stay without compromising oncologic outcomes. Am J Surg (2015) 209(3):557–63. doi: 10.1016/j.amjsurg.2014.11.001 25596756

[4] Shin SH Kim SC Song KB Hwang DW Lee JH Lee D . A comparative study of laparoscopic vs open distal pancreatectomy for left-sided ductal adenocarcinoma: A propensity score-matched analysis. J Am Coll Surgeons (2015) 220(2):177–85. doi: 10.1016/j.jamcollsurg.2014.10.014 25529901

[5] Venkat R Edil BH Schulick RD Lidor AO Makary MA Wolfgang CL . Laparoscopic distal pancreatectomy is associated with significantly less overall morbidity compared to the open technique a systematic review and meta-analysis. Ann Surg (2012) 255(6):1048–59. doi: 10.1097/SLA.0b013e318251ee09 22511003

[6] Melvin WS Needleman BJ Krause KR Ellison EC . Robotic resection of pancreatic neuroendocrine tumor. J Laparoendosc Adv Surg Tech A (2003) 13(1):33–6. doi: 10.1089/109264203321235449 12676019

[7] Hong S Song KB Madkhali AA Hwang K Yoo D Lee JW . Robotic versus laparoscopic distal pancreatectomy for left-sided pancreatic tumors: A single surgeon's experience of 228 consecutive cases. Surg Endosc (2020) 34(6):2465–73. doi: 10.1007/s00464-019-07047-8 31463719

[8] Shakir M Boone BA Polanco PM Zenati MS Hogg ME Tsung A . The learning curve for robotic distal pancreatectomy: An analysis of outcomes of the first 100 consecutive cases at a high-volume pancreatic centre. Hpb (2015) 17(7):580–6. doi: 10.1111/hpb.12412 PMC4474504 25906690

[9] Benizri EI Germain A Ayav A Bernard JL Zarnegar R Benchimol D . Short-term perioperative outcomes after robot-assisted and laparoscopic distal pancreatectomy. J Robot Surg (2014) 8(2):125–32. doi: 10.1007/s11701-013-0438-8 27637522

[10] Moher D Liberati A Tetzlaff J Altman DG . Preferred reporting items for systematic reviews and meta-analyses: The PRISMA statement. Int J Surg (2010) 8(5):336–41. doi: 10.1016/j.ijsu.2010.02.007 20171303

[11] Lo CK Mertz D Loeb M . Newcastle-Ottawa Scale: Comparing reviewers' to authors' assessments. BMC Med Res Methodol (2014) 14:45. doi: 10.1186/1471-2288-14-45 24690082 PMC4021422

[12] Hozo SP Djulbegovic B Hozo I . Estimating the mean and variance from the median, range, and the size of a sample. BMC Med Res Methodol (2005) 5:13. doi: 10.1186/1471-2288-5-13 15840177 PMC1097734

[13] Adam MA Choudhury K Goffredo P Reed SD Blazer D 3rd Roman SA . Minimally Invasive Distal Pancreatectomy for Cancer: Short-Term Oncologic Outcomes in 1733 Patients. World J Surg (2015) 39(10):2564–72. doi: 10.1007/s00268-015-3138-x 26154576

[14] Chen S Zhan Q Chen JZ Jin JB Deng XX Chen H . Robotic approach improves spleen-preserving rate and shortens postoperative hospital stay of laparoscopic distal pancreatectomy: a matched cohort study. Surg Endosc (2015) 29(12):3507–18. doi: 10.1007/s00464-015-4101-5 25791063

[15] Lee SY Allen PJ Sadot E D'Angelica MI DeMatteo RP Fong Y . Distal Pancreatectomy: A Single Institution's Experience in Open, Laparoscopic, and Robotic Approaches. J Am Coll Surgeons (2015) 220(1):18–27. doi: 10.1016/j.jamcollsurg.2014.10.004 25456783

[16] Liu R Liu Q Zhao ZM Tan XL Gao YX Zhao GD . Robotic versus laparoscopic distal pancreatectomy: A propensity score-matched study. J Surg Oncol (2017) 116(4):461–9. doi: 10.1002/jso.24676 28628713

[17] Xourafas D Ashley SW Clancy TE . Comparison of perioperative outcomes between open, laparoscopic, and robotic distal pancreatectomy: An analysis of 1815 patients from the ACS-NSQIP procedure-targeted pancreatectomy database. J Gastrointest Surg (2017) 21(9):1442–52. doi: 10.1007/s11605-017-3463-5 28573358

[18] Zhang JQ Jin JB Chen S Gu J Zhu Y Qin K . Minimally invasive distal pancreatectomy for PNETs: laparoscopic or robotic approach? Oncotarget (2017) 8(20):33872–83. doi: 10.18632/oncotarget.17513 PMC5464919 28477012

[19] Liu Q Zhao ZM Tan XL Yuanxing G Yong X Rong L . Short- and mid-term outcomes of robotic versus laparoscopic distal pancreatosplenectomy for pancreatic ductal adenocarcinoma: A retrospective propensity score-matched study. Int J Surg (2018) 55:81–6. doi: 10.1016/j.ijsu.2018.05.024 29802919

[20] Raoof M Nota CLMA Melstrom LG Warner SG Woo Y Singh G . Oncologic outcomes after robot-assisted versus laparoscopic distal pancreatectomy: Analysis of the National Cancer Database. J Surg Oncol (2018) 118(4):651–6. doi: 10.1002/jso.25170 PMC6386178 30114321

[21] Lyman WB Passeri M Sastry A Cochran A Iannitti DA Vrochides D . Robotic-assisted versus laparoscopic left pancreatectomy at a high-volume, minimally invasive center. Surg Endosc (2019) 33(9):2991–3000. doi: 10.1007/s00464-018-6565-6 30421076

[22] Marino MV Mirabella A Gomez Ruiz M Komorowski AL . Robotic-assisted versus laparoscopic distal pancreatectomy: The results of a case-matched analysis from a tertiary care center. Dig Surg (2020) 37(3):229–39. doi: 10.1159/000501428 31269490

[23] De Pastena M Esposito A Paiella S Surci N Montagnini G Marchegiani G . Cost-effectiveness and quality of life analysis of laparoscopic and robotic distal pancreatectomy: A propensity score-matched study. Surg Endosc (2021) 35(3):1420–8. doi: 10.1007/s00464-020-07528-1 32240383

[24] Lof S van der Heijde N Abuawwad M Al-Sarireh B Boggi U Butturini G . Robotic versus laparoscopic distal pancreatectomy: Multicentre analysis. Brit J Surg (2021) 108(2):188–95. doi: 10.1093/bjs/znaa039 33711145

[25] Di Franco G Peri A Lorenzoni V Palmeri M Furbetta N Guadagni S . Minimally invasive distal pancreatectomy: A case-matched cost-analysis between robot-assisted surgery and direct manual laparoscopy. Surg Endosc (2022) 36(1):651–62. doi: 10.1007/s00464-021-08332-1 PMC8741657 33534074

[26] Kwon J Lee JH Park SY Park Y Lee W Song KB . A comparison of robotic versus laparoscopic distal pancreatectomy: Propensity score matching analysis. Int J Med Robot. (2022) 18(2):e2347. doi: 10.1002/rcs.2347 34726827

[27] Dindo D Demartines N Clavien PA . Classification of surgical complications: A new proposal with evaluation in a cohort of 6336 patients and results of a survey. Ann Surg (2004) 240(2):205–13. doi: 10.1097/01.sla.0000133083.54934.ae PMC1360123 15273542

[28] Kamarajah SK Sutandi N Robinson SR French JJ White SA . Robotic versus conventional laparoscopic distal pancreatic resection: A systematic review and meta-analysis. HPB (Oxford) (2019) 21(9):1107–18. doi: 10.1016/j.hpb.2019.02.020 30962137

[29] Magistri P Boggi U Esposito A Carrano FM Pesi B Ballarin R . Robotic vs open distal pancreatectomy: A multi-institutional matched comparison analysis. J Hepatobiliary Pancreat Sci (2021) 28(12):1098–106. doi: 10.1002/jhbp.881 33314791

[30] Wang M Zhang H Wu Z Zhang Z Peng B . Laparoscopic pancreaticoduodenectomy: single-surgeon experience. Surg Endosc (2015) 29(12):3783–94. doi: 10.1007/s00464-015-4154-5 25783837

[31] Croome KP Farnell MB Que FG Reid-Lombardo KM Truty MJ Nagorney DM . Total laparoscopic pancreaticoduodenectomy for pancreatic ductal adenocarcinoma: Oncologic advantages over open approaches? Ann Surg (2014) 260(4):633–8. doi: 10.1097/SLA.0000000000000937 25203880

[32] Huang B Feng L Zhao J . Systematic review and meta-analysis of robotic versus laparoscopic distal pancreatectomy for benign and malignant pancreatic lesions. Surg Endosc (2016) 30(9):4078–85. doi: 10.1007/s00464-015-4723-7 26743110

[33] Napoli N Kauffmann EF Perrone VG Miccoli M Brozzetti S Boggi U . The learning curve in robotic distal pancreatectomy. Updates Surg (2015) 67(3):257–64. doi: 10.1007/s13304-015-0299-y 25990666

[34] Shyr BU Chen SC Shyr YM Wang SE . Learning curves for robotic pancreatic surgery-from distal pancreatectomy to pancreaticoduodenectomy. Med (Baltimore) (2018) 97(45):e13000. doi: 10.1097/MD.0000000000013000 PMC6250552 30407289

[35] Al Abbas AI Wang C Hamad AB Knab LM Rice MK Moser AJ . Mentorship and formal robotic proficiency skills curriculum improve subsequent generations' learning curve for the robotic distal pancreatectomy. HPB (Oxford) (2021) 23(12):1849–55. doi: 10.1016/j.hpb.2021.04.022 34059420

[36] Takahashi C Shridhar R Huston J Meredith K . Outcomes associated with robotic approach to pancreatic resections. J Gastrointest Oncol (2018) 9(5):936–41. doi: 10.21037/jgo.2018.08.04 PMC6219977 30505596

[37] Guerrini GP Lauretta A Belluco C Olivieri M Forlin M Basso S . Robotic versus laparoscopic distal pancreatectomy: An up-to-date meta-analysis. BMC Surg (2017) 17(1):105. doi: 10.1186/s12893-017-0301-3 29121885 PMC5680787

[38] Feng Q Xin Z Zhu B Liao M Liao W Zeng Y . Perioperative and short-term oncological outcomes following laparoscopic versus open pancreaticoduodenectomy after learning curve in the past 10 years: a systematic review and meta-analysis. Gland Surg (2021) 10(5):1655–68. doi: 10.21037/gs-20-916 PMC8184374 34164310

